# Concomitant sensory stimulation during therapy to enhance hand functional recovery post stroke

**DOI:** 10.1186/s13063-022-06241-9

**Published:** 2022-04-05

**Authors:** Na Jin Seo, Viswanathan Ramakrishnan, Michelle L. Woodbury, Leonardo Bonilha, Christian Finetto, Christian Schranz, Gabrielle Scronce, Kristen Coupland, Jenna Blaschke, Adam Baker, Keith Howard, Caitlyn Meinzer, Craig A. Velozo, Robert J. Adams

**Affiliations:** 1 Department of Rehabilitation Sciences, Department of Health Science and Research, Medical University of South Carolina, 151B Rutledge Ave, MSC 962, Charleston, SC 29425 USA; 2 Ralph H. Johnson VA Medical Center, Charleston, SC USA; 3 Department of Health Science and Research, Medical University of South Carolina, 77 President St, MSC 700, Charleston, SC 29425 USA; 4 Department of Public Health Sciences, Medical University of South Carolina, 135 Cannon St, Charleston, SC 29425 USA; 5 Department of Neurology, Medical University of South Carolina, 96 Jonathan Lucas St, MSC 606, Charleston, SC 29425 USA

**Keywords:** Stroke, Upper extremity, Physical rehabilitation, Hand function, Subliminal stimulation, Physical stimulation, Stroke rehabilitation, Neurologic rehabilitation, Occupational therapy, Upper limb paresis, Hand, EEG, Paralysis, Randomized controlled trial

## Abstract

**Background:**

Post-stroke hand impairment is prevalent and persistent even after a full course of rehabilitation. Hand diminishes stroke survivors’ abilities for activities of daily living and independence. One way to improve treatment efficacy is to augment therapy with peripheral sensory stimulation. Recently, a novel sensory stimulation, TheraBracelet, has been developed in which imperceptible vibration is applied during task practice through a wrist-worn device. The objective of this trial is to determine if combining TheraBracelet with hand task practice is superior to hand task practice alone.

**Methods:**

A double-blind randomized controlled trial will be used. Chronic stroke survivors will undergo a standardized hand task practice therapy program (3 days/week for 6 weeks) while wearing a device on the paretic wrist. The device will deliver TheraBracelet vibration for the treatment group and no vibration for the control group. The primary outcome is hand function measured by the Wolf Motor Function Test. Other outcomes include the Box and Block Test, Action Research Arm Test, upper extremity use in daily living, biomechanical measure of the sensorimotor grip control, and EEG-based neural communication.

**Discussion:**

This research will determine clinical utility of TheraBracelet to guide future translation. The TheraBracelet stimulation is delivered via a wrist-worn device, does not interfere with hand motion, and can be easily integrated into clinical practice. Enhancing hand function should substantially increase stroke survivors' independence and quality of life and reduce caregiver burden.

**Trial registration:**

NCT04569123. Registered on September 29, 2020

## Administrative information

Note: the numbers in curly brackets in this protocol refer to SPIRIT checklist item numbers. The order of the items has been modified to group similar items (see http://www.equator-network.org/reporting-guidelines/spirit-2013-statement-defining-standard-protocol-items-for-clinical-trials/).

**Table:** 

Title {1}	Concomitant sensory stimulation during therapy to enhance hand functional recovery post stroke
Trial registration {2a and 2b}.	NCT04569123. Clinicaltrials.gov
Protocol version {3}	#6. 7/28/2021
Funding {4}	NIH/NICHD 1R01HD094731-01A1
Author details {5a}	Na Jin Seo, PhD, Department of Rehabilitation Sciences, Department of Health Science and Research, Medical University of South Carolina, Charleston, SC, USA. Ralph H. Johnson VA Medical Center, Charleston, SC, USA. ORCID 0000-0001-6446-5905. seon@musc.edu. 151B Rutledge Ave, MSC 962, Charleston, SC 29425. 843-792-0084. Viswanathan Ramakrishnan, PhD, Department of Public Health Sciences, Medical University of South Carolina, Charleston, SC, USA. ORCID 0000-0002-4098-0539. ramakris@musc.edu. 135 Cannon St, Charleston, SC 29425. 843-876-1153. Michelle L. Woodbury, PhD, OTR/L, Department of Health Science and Research, Medical University of South Carolina, Charleston, SC, USA. 0000-0001-9289-570X. woodbuml@musc.edu. 77 President St, MSC 700, Charleston, SC 29425. 843-792-1671. Leonardo Bonilha, MD, PhD, Department of Neurology, Medical University of South Carolina, Charleston, SC, USA. bonilha@musc.edu. 96 Jonathan Lucas St, MSC 606, Charleston, SC 29425. 843-876-8311. Christian Finetto, PhD, Department of Health Sciences and Research, Medical University of South Carolina, Charleston, SC, USA. ORCID 0000-0003-0520-2034. finetto@musc.edu. 77 President St, MSC 700, Charleston, SC 29425. 843-792-5645. Christian Schranz, PhD, Department of Health Sciences and Research, Medical University of South Carolina, Charleston, SC, USA. ORCID: 0000-0003-1102-7180. schranz@musc.edu. 77 President St, MSC 700, Charleston, SC 29425. Gabrielle Scronce, PT, DPT, PhD, Department of Health Sciences and Research, Medical University of South Carolina, Charleston, SC, USA. ORCID 0000-0002-3861-1371. scronce@musc.edu. 77 President St, MSC 700, Charleston, SC 29425. Kristen Coupland, MS, OTR/L, CSRS, CDRS, Department of Health Sciences and Research, Medical University of South Carolina, Charleston, SC, USA. coupland@musc.edu. 77 President St, MSC 700, Charleston, SC 29425. Jenna Blaschke, BA, Division of Occupational Therapy, Department of Rehabilitation Sciences, Medical University of South Carolina, Charleston, SC, USA. blaschkj@musc.edu. 151B Rutledge Ave, MSC 962, Charleston, SC 29425. 803-459-6403. Adam Baker, BS, Department of Health Sciences and Research, Medical University of South Carolina, Charleston, SC, USA. bakerdon@musc.edu. 77 President St., MSC 700, Charleston, SC 29425 Keith Howard, BS, Department of Health Sciences and Research, Medical University of South Carolina, Charleston, SC, USA. howardke@musc.edu. 77 President St, MSC 700, Charleston, SC 29425. 843-792-2917. Caitlyn Meinzer, PhD, Department of Public Health Sciences, Medical University of South Carolina, Charleston, SC, USA. ellerbcn@musc.edu. 135 Cannon St, Charleston, SC 29425. 843-792-6588. Craig A. Velozo, PhD, Division of Occupational Therapy, Department of Rehabilitation Sciences, Medical University of South Carolina, Charleston, SC, USA. velozo@musc.edu. 151B Rutledge Ave, MSC 962, Charleston, SC 29425. 803-459-6403. Robert J. Adams, MD, Department of Neurology, Medical University of South Carolina, Charleston, SC, USA. adamsrj@musc.edu. 96 Jonathan Lucas St, MSC 606, Charleston, SC 29425. 843-792-3020.
Name and contact information for the trial sponsor {5b}	Medical University of South Carolina, College of Health Professions 843-792-3328, 151B Rutledge Ave, Charleston, SC, 29425 NIH/NICHD, 1-800-370-2943 Email: NICHDInformationResourceCenter@mail.nih.gov Fax: 1-866-760-5947 Mail: P.O. Box 3006, Rockville, MD, 20847
Role of sponsor {5c}	Oversight for regulatory requirements.

## Introduction

### Background and rationale {6a}

Stroke is a leading cause of long-term disability in the USA [1]. More than two thirds of nearly 7 million stroke survivors in the USA [1] have persistent hand impairment even after a full course of rehabilitation [2]. Hand impairment diminishes stroke survivors’ abilities for activities of daily living including self-care, hygiene, leisure, and employment, which lowers their independence and increases caregiver burdens [3, 4]. Due to increasing limitations on access to therapy, improving the efficacy of rehabilitation treatment to improve hand function is of significant importance.

Recent meta-analysis shows that upper extremity motor function improves more when therapy is augmented by peripheral sensory stimulation, compared with therapy alone [5]. The scientific rationale is that afferent input is a powerful driver of change in the motor cortex [6, 7]. Specifically, direct projections from the cortical hand sensory areas to motor areas are evidenced in intracortical microstimulation studies in animals [8–11] and in the long-latency cutaneomuscular reflex in humans [12, 13]. These direct projections from the sensory to motor cortex enables afferent sensory stimulation to directly affect motor output [14–16]. For example, corticomotoneuronal excitability as measured using transcranial magnetic stimulation (TMS) has been shown to increase during muscle vibration, compared to no vibration [17, 18]. After 30 min of electrical stimulation of the whole hand via a mesh glove, functional magnetic resonance imaging blood-oxygen-level-dependent signals during finger movement in the primary motor and somatosensory area increased, compared to that before the stimulation [19]. After 2 h of transcutaneous electrical nerve stimulation (TENS), corticomotoneuronal excitability increases via GABAergic mechanism [20]. Daily 1-h TENS over 3 weeks led to increased cortical motor map area and volume of involved muscles (assessed using TMS) [21].

Based on this framework, peripheral sensory stimulation has been used in conjunction with therapy to increase neuroplasticity and motor recovery more than therapy alone in patients with neurologic motor impairment [22–34]. Specifically, 30-min vibration to the upper limb muscles followed by 1-h physiotherapy, repeated for 3 consecutive days, resulted in greater improvement in the Wolf Motor Function Test (WMFT) than dose-matched physiotherapy alone, which sustained 2 weeks after the intervention (*n* = 30 chronic stroke survivors, randomized to *n* = 15/group) [24]. This greater functional improvement was associated with greater corticomotor excitability and motor map areas (assessed using TMS) for the stimulation + therapy group compared with the therapy only group [24]. In another study, 2-h TENS followed by 4-h task practice therapy, repeated for 10 days, resulted in greater improvements in the Fugl-Meyer Upper Extremity Assessment and Action Research Arm Test (ARAT) than dose-matched therapy alone, which sustained at 1-month follow-up (*n* = 36 chronic stroke survivors randomized to *n* = 18/group) [29]. Meta-analysis supports this premise of using sensory stimulation to augment motor recovery [5].

Unfortunately, most modalities of peripheral sensory stimulation interfere with natural hand tasks. Specifically, suprathreshold stimulation causes sensation irrelevant to tasks at hand, including TENS-induced tingling sensation [19, 21]. Wearing of a glove or a finger cap hampers dexterous finger movement and causes a sense of discomfort [35, 36]. Thus, most modalities of sensory stimulation are administered prior to each therapy session while a person is in a sedentary posture, requiring additional time commitment ranging from 30 min to 2 h a day [22–30]. These constraints make it difficult for implementation and patient adherence to a stimulation regimen [37]. Furthermore, the effect diminishes 20–30 min after the stimulation [20, 38], weakening its effect during therapy. Studies that used sensory stimulation *during* therapy had no control group (with no stimulation) [31, 32] or did not test stroke survivors [33, 34] and used suprathreshold stimulation causing sensation irrelevant to tasks.

To address this practical limitation of the current sensory stimulation method and fully leverage the therapeutic benefits of sensory stimulation, we have developed an innovative sensory stimulation: “TheraBracelet” is imperceptible random-frequency vibration applied to wrist skin via a wrist-worn device. TheraBracelet does not interfere with natural hand tasks since the stimulation is imperceptible and delivered via a device worn on the wrist [39–41]. The theoretical framework is that imperceptible random-frequency vibration stimulates mechanoreceptors in wrist skin and afferents [42], adds small random currents to neurons in the sensorimotor cortex, triggering coherent [43] firing [44–46] at the peak of inputs related to hand tasks, and consequently enhances neural communication [47, 48] for hand tasks [49–52] and functional recovery. Pilot studies showed preliminary efficacy [53, 54].

A randomized controlled trial to determine clinical utility of TheraBracelet is described herein. This research is expected to result in an efficacious therapeutic adjunct to enhance upper extremity rehabilitation outcomes for people with stroke. Enhancing hand motor function and therapy outcome with TheraBracelet is expected to contribute to improving stroke survivors’ abilities to perform activities of daily living, increasing functional independence, and reducing caregiver burden, thereby leading to increased quality of life for both stroke survivors and caregivers.

### Objectives {7}

The objective of this study is to determine if combining TheraBracelet with hand task practice is superior to hand task practice alone in an adequately powered study.

### Trial design {8}

The trial design is a double-blind randomized controlled trial involving two parallel groups. Participants will be randomly assigned to either the experimental or control group. Half will be in the experimental group and the other half will be in the control group. Both groups will undergo standardized hand task practice therapy while wearing a device on the paretic wrist. The device will deliver TheraBracelet vibration for the treatment group and no vibration for the control group during therapy. The superiority of the experimental condition over the control condition will be examined.

## Methods: participants, interventions, and outcomes

### Study setting {9}

The study setting is the research laboratory of the Medical University of South Carolina, Charleston, SC, USA.

### Eligibility criteria {10}

Inclusion criteria:

- Adult (> = 18 years old)
- Survived a stroke at least 6 months ago
- WMFT [55] total average time > 10 s
- WMFT hand task average time < 120 s

Exclusion criteria:

- Concurrent upper limb therapy
- Change in spasticity medication or botulinum toxin injection in the upper limb within 3 months prior to or during enrollment
- Severe spasticity (Modified Ashworth Scale [56] = 4–5 out of 5) that prohibits engagement in task practice
- Comorbidity such as complete upper extremity deafferentation, orthopedic conditions limiting motion [57], premorbid neurologic conditions, or compromised skin integrity of the wrist due to burn or long-term use of blood thinners
- Language barrier or cognitive impairment that precludes following instructions and/or providing consent

### Who will take informed consent? {26a}

Research staff approved by the Institutional Review Board (IRB) will take informed consent. The consent process will take place in a private room when the potential participant comes to the laboratory on a scheduled time agreed upon between the study personnel and the participant. The content of the consent will be verbally explained to the participant and the participant will be asked to raise any questions and concerns.

### Additional consent provisions for collection and use of participant data and biological specimens {26b}

The consent includes sharing of de-identified data with the public and other investigators in publications and ClinicalTrials.gov. In addition, authorization for use and release of individually identifiable health information collected for research with the Medical University of South Carolina IRB and the funding agency will be obtained in writing. This trial does not involve collecting biological specimens for storage.

### Interventions

#### Explanation for the choice of comparators {6b}

The control group will receive standardized task practice therapy while receiving no vibration from the device on the paretic wrist. The control condition represents the standard task practice therapy [58] without the TheraBracelet stimulation. For both groups, the device is worn during therapy only (not outside therapy).

#### Intervention description {11a}

*Intervention schedule*: All participants will undergo 6 weeks of standardized task practice therapy. The therapy schedule will be 3 sessions/week for 6 weeks, with each session lasting 1–2 h to complete 300 movement repetitions. This therapy schedule is chosen to simulate the outpatient rehabilitation model [59] and facilitate potential translation of the protocol for implementation in rehabilitation practice. This therapy schedule is also similar with other stroke upper limb rehabilitation programs with distributed practice schedules, enabling comparisons [60–64].

*Task practice therapy*: Therapists will be trained to administer the task practice therapy according to standardized procedures. The treatment manual, containing a menu of task practice activities, was developed by two experienced occupational therapists, based on the EXCITE trial [65] manual and Dr. Lang’s Task Specific Practice text [58]. The main principles of the therapy program are detailed below.

**Fig. 1:**
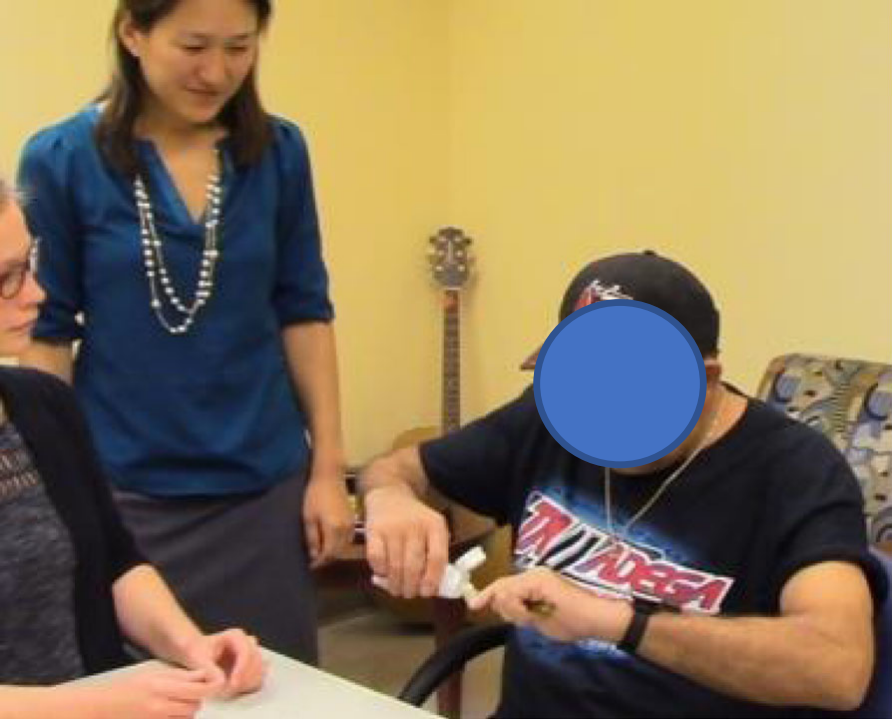
An example therapy session with the wrist-worn device that can deliver TheraBracelet stimulation

1. Specificity: All therapy tasks address hand/finger motions, because TheraBracelet has been shown to impact hand/finger control in our preliminary studies [51, 53]. Selected tasks naturally require several repetitions of the specified motion.
2. Structure: For organizational purposes, the tasks are categorized into self-care, household care, leisure, and vocation. Each category contains two types of tasks: tasks requiring (i) primarily in-hand manipulation and (ii) reaching. The in-hand manipulation tasks will be performed within the subject’s reachable workspace (e.g., in front of the torso) to exclusively focus on hand dexterity which is the intervention target using TheraBracelet. The reaching tasks will be performed by reaching to grasp/place objects throughout the reachable workspace to integrate hand dexterity skills with the proximal upper limb motion to maximize the functional relevance of the treatment. A total of 4 tasks (2 in-hand and 2 reaching, from each of 2 categories of choice) will be practiced in each session. Repetitions for in-hand vs. reaching will be balanced.
3. Saliency: To assure the task practice is meaningful, thus motivating for the subject, the therapist and subject will work together to select the tasks from the task menu. This maximizes the potential for the subject to apply skills learned in clinic to their real world tasks and goals [58].
4. Repetitions: To standardize therapy dosage, we will aim for each participant to achieve 300 movement repetitions per session. This dosage is feasible in a 1–2 h timeframe [61, 66] with no excessive pain or fatigue [60], results in functional improvements [61], and corresponds to the lower end of the repetitions in animal and motor learning studies that have been shown to promote neural plasticity and behavioral changes [67, 68]. One repetition will be defined for each activity in the task manual to ensure consistency in counting repetitions. For in-hand tasks, one repetition will be defined as one hand manipulation (e.g., place three coins in the paretic palm, and transfer to fingertips and insert into a slot with one coin at a time for 3 repetitions). For reaching tasks, one repetition will be reach-grasp-manipulate-release [61] (e.g., reach for/grasp a toothbrush, apply toothpaste, simulate brushing motions four times, and replace the toothbrush on the table, Fig. 1).
5. Difficulty: Brain reorganization occurs through motor learning that requires problem solving, not simple repetition of mastered skills, as shown in human [69, 70] and animal studies [67, 71, 72]. Thus, tasks will be at a “just-right” difficulty level that does not overwhelm or underwhelm participants. Specifically, we will implement tasks such that the participant practices movements that s/he can partially perform, as opposed to easily perform or cannot perform. We will increase or decrease difficulty based on the participant’s success rate of the task (≥ 90% or ≤ 50%), task pace, and patient input [58] following the Challenge Point Framework [73]. Grading of tasks will be accomplished by changing the object weight, size [74–76], shape [77, 78], slipperiness [79–84], compliance, stability [85], and location, use of adaptive materials (e.g., nonslip mat to prevent items from moving), task complexity, and speed and accuracy of movement.

*TheraBracelet stimulation*: TheraBracelet is imperceptible random-frequency vibration applied to wrist skin via a wrist-worn device. TheraBracelet stimulation parameters were determined based on literature and our previous data as detailed below.

1. Frequency: The vibrator will be driven by random-frequency signal low-pass filtered at 500 Hz for the following reasons. Literature in stochastic resonance collectively demonstrates that broadband random-frequency (white noise) stimulation enhances neural communication and signal detection (see reviews [43, 44, 86] and applications [47, 49, 87–92]). The benefit of the random-frequency stimulation compared to constant frequency stimulation is that it is less subject to desensitization (or sensory habituation). Literature comparing the two types of stimulation shows that temporally non-uniform stimulation improves tactile sensation and modulates the central nervous system excitability, while such effects could not be obtained with constant frequency stimulation [93, 94]. By preventing desensitization, TheraBracelet is expected to provide a benefit that persists throughout therapy sessions. As for the bandwidth, existing evidence shows that one’s ability to detect 25 Hz signal was enhanced when accompanied by random-frequency vibration bandwidth filtered at 0.125–50 Hz, and similarly, ability to detect 250 and 400 Hz signal was enhanced with random-frequency vibration of 50–500 Hz [87]. For various sensorimotor tasks including hand grip, touch detection, and texture discrimination, random-frequency vibration low-pass filtered at 300 Hz (i.e., 0–300 Hz) was used to successfully enhance sensorimotor performance [89]. Since human mechanoreceptors can detect vibration from 0.4 to 500 Hz [95], we decided to use random-frequency vibration low-pass filtered at 500 Hz to span the entire frequency spectrum for human mechanoreceptors.
2. Location: The vibration will be applied to the dorsal wrist, because the same beneficial effects on improved detection of fingertip touch during vibration vs. no vibration were obtained for all vibration locations we tested—volar wrist, dorsal wrist, dorsum of the hand near the first and the second knuckle, thenar area, and hypothenar area, in chronic stroke survivors [50] as well as healthy adults [52]. The wrist is optimal for a wearable device, as the device can be worn on the wrist similar to wearing a wristwatch.
3. Intensity: Vibration intensity 60% of the sensory threshold (imperceptible) will be used, because that intensity enhanced finger touch detection the most (as assessed by the monofilament test [96]), while 40% and 80% of the sensory threshold had lesser effects [50, 97]. Further, vibration 120% of the sensory threshold (perceptible) degraded finger sensation [52]. This intensity-effect relationship is consistent with the bell-shaped curve (i.e., inverted U-shape function) reported in stochastic resonance literature in which vibration intensities at 33–67% of the sensory threshold improved sensory perception to a greater extent than did intensities of 0%, 83%, and 100% of the sensory threshold [87], and suprathreshold vibration degraded sensation [88]. These results suggest that vibration intensity should be high enough to facilitate signal transmission but not too high to mask other signals [87].

For both groups, the participant’s sensory threshold will be determined at the beginning of each therapy session using a custom-developed software program that utilizes the staircase method [98]. Specifically, the vibration amplitude will be increased or decreased until the participant verbally indicates that s/he could or could not perceive the wrist vibration, respectively. The threshold will be determined as the average of 6 amplitudes that the participant could barely feel.

For the treatment group, TheraBracelet stimulation will be turned on when movement is detected at or higher than the acceleration magnitude that corresponds to movement of one finger only. Upon movement detection, TheraBracelet stimulation will be turned on for 1 s, after which the stimulation will continue if movement is detected again, or stop if no movement is detected, such that stimulation will be on during all movements but not for more than 1 s after the movement ends to minimize potential habituation to the stimulation. The consistency in the frequency characteristics from the device will be verified weekly using a laser vibrometer (OMS Corp, Laguna Hills, CA, USA).

*Transfer package*: To achieve greater independence at home using the participant’s improved motor capacities from therapy, we will implement a transfer package [99]. Transfer package is adopted from the behavioral intervention field specifically for physical neurorehabilitation, based on the principles of self-monitoring [100, 101], contracting/negotiating for specific behaviors [102], phone contacts to increase compliance [103–105], and problem-solving to overcome barriers [106–110]. Components of the transfer package are detailed below.

1. Behavioral contracts: The researcher will negotiate a contract with the participant and separately with the caregiver, if any, in which they agree to comply with the intervention including completion of all assignments and using the paretic hand on specific activities of daily living as much as possible outside the lab. The negotiated document will be signed by the participant, caregiver, and researcher to emphasize the character of the document as a contract.
2. Activity tracking: The Motor Activity Log (MAL) [111] will be administered at baseline and reviewed weekly (in person during 6-week intervention and in weekly phone contacts following intervention) to track amount and quality of paretic hand use in daily living and to keep participants’ attention on use of the paretic hand at home.
3. Problem solving: The therapist will help participants think through barriers to using the paretic hand at home and ways to overcome them (e.g., use modified tools, ensure safe environment) at each visit during the 6-week intervention and in weekly phone contacts following intervention.
4. Daily home diary: Specific activities that the participant can try using the paretic hand at home will be determined and written down. The participant will record how many times s/he has used the paretic hand for the specified activity.
5. Home skill assignment: Participants will be assigned to perform 4 specific tasks with the paretic hand daily with specified repetitions and record on a written check-off sheet during 6-week intervention. The tasks will be chosen to improve the participant’s most significant movement deficits. Following intervention, participants will be prescribed a written individualized home skill practice program entailing specific tasks to practice with specified repetitions for each day of the week for 4 weeks. When noncompliance is indicated for the daily home diary and home skill assignment, the therapist will inquire into the reasons and problem-solve with the participant/caregiver on how to reverse the trend.

#### Criteria for discontinuing or modifying allocated interventions {11b}

Participants with repeated no shows will be withdrawn.

#### Strategies to improve adherence to interventions {11c}

The intervention requires in-person visit to the laboratory. Support for parking and transportation assistance will be provided as necessary. The visit schedule will be printed and handed out to each participant. Reminder phone calls will be made. A waiting room will be provided within the building for caregivers. Remuneration for participation will be provided. All COVID-19 precautions will be taken to ensure health and safety of participants and study personnel.

#### Relevant concomitant care permitted or prohibited during the trial {11d}

Concurrent upper limb rehabilitation is prohibited during participation in the study. Change in spasticity medication or botulinum toxin injection in the upper limb is prohibited during the study. Concomitant care for other issues is permitted.

#### Provisions for post-trial care {30}

Participants will be followed up until 1 month post intervention for adverse events. Necessary medical treatment will be participants’ responsibilities.

#### Outcomes {12}

The primary outcome measure is hand function measured by the WMFT time [55]. The primary time point is from the baseline to 1-month follow-up. Hand function will also be assessed using the Box and Block Test (BBT) [112] and ARAT [113]. Paretic upper limb use in daily living will be measured using accelerometers [114] and MAL [111]. The patient-centered outcome measures of the Stroke Impact Scale, perceived meaningfulness of the intervention [115], and usability feedback on TheraBracelet will also be obtained. Other outcome measures will include sensorimotor grip control and neural communication for hand grip, quantified as the digit force directional control [116, 117] and EEG connectivity in the cortical sensorimotor network [118].

#### Participant timeline {13}

**Fig. 2:**
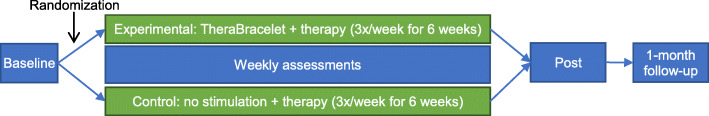
Participation timeline. All participants will have a baseline assessment, 6 weeks of task practice therapy with weekly hand function assessments, post assessment, and 1-month follow-up assessment

The participant timeline is shown in Fig. 2. A baseline assessment will take place prior to the intervention. The intervention will entail a total of 18 task practice sessions with or without TheraBracelet stimulation over 6 weeks. Hand function assessments will be performed weekly during the 6-week intervention to examine the pattern of progress. The post assessment will take place within a week from the last intervention session. The 1-month follow-up is to assess retention.

#### Sample size {14}

The primary objective is to determine clinical potential of TheraBracelet as measured by WMFT time. Insight on its minimal clinically important difference (MCID) is available in literature. Since the minimum detectable change ranges from 1.0 to 3.4 s [119], it could be interpreted that greater than 3.4 s change represents true clinical change. Anchor-based and distribution-based minimum detectable change and MCID range from 1.5 to 4.36 s [120]. Lang et al. (2008) report change of 19 ± 16 s was perceived as meaningful by acute stroke survivors, while 4 ± 7 s was not perceived as meaningful for the dominant hand [115]. Variability was high in that study, where patients’ perception of meaningfulness ranged between 3 and 11 s. In a recent ICARE trial [62], improvement in WMFT time of 8 ± 3 s after inpatient rehabilitation for acute stroke survivors (1.5 month post stroke) was associated with improvement in Stroke Impact Scale hand portion of 37 ± 5 (in percent points) which substantially exceeded MCID of 25 points (representing easement for tasks by a full category, e.g., from “very difficult” to “somewhat difficult”) [121]. Based on these, 4 to 7 s between-group difference in improvement in WMFT time will be considered minimally clinically meaningful.

This study will be powered to detect sustained difference at follow-up of at least 4 s in WMFT between the treatment and control groups. For the repeated measures design with a significance level of 0.017 (adjusted for simultaneously comparing 3 outcomes), at least 80% power, and 4 time points, for a standard deviation (SD) of 5.1 s and compound symmetry correlation of 0.92 (both based on our pilot study [53]), sample size of 32 per group will be adequate. We believe this is a very conservative estimate since we used data from a 2-week pilot intervention [53] to compute power, and this 6-week intervention is expected to yield greater effects [54]. Adjusting for 15% attrition, 38 per group is planned (total *n* = 76).

This sample size is sufficient for other outcome measures at follow-up by allowing detection of the minimum between-group difference in (i) BBT of 5.3 (with SD = 4.9 from our pilot study [53]) which is approximately the minimum detectable change of 5.5 [122], (ii) digit force directional control of 4.9° (SD = 4.54) which is approximately the group difference at follow-up observed in our pilot study and distinguishes grip control impairment levels [116], and (iii) EEG connectivity of 0.017 (SD = 0.016) which associates with psychophysical behavior change [123].

#### Recruitment {15}

Participants will be recruited from the MUSC Registry for Stroke Recovery (RESTORE). Currently, the RESTORE has information of more than a thousand stroke survivors who have agreed to be contacted for research (approved MUSC IRB PRO# 37803). The registry is growing every day, as approximately 500 new stroke cases are treated at the MUSC Stroke Center every year. All eligible stroke patients in the inpatient stroke units as well as the outpatient clinic are contacted by a dedicated recruiter supported by the NIH-funded Center of Biomedical Excellence for Stroke Recovery at MUSC to be enrolled in the registry. In addition to recruiting stroke survivors from the hospital, we recruit stroke survivors from the community by having a dedicated outreach therapist from the center visit local stroke support group meetings and develop relationships with stroke survivors, caregivers, and clinicians, and also by organizing community outreach events such as stroke caregiver summits and stroke recovery community engagement, to establish grassroots connections with stroke survivors and caregivers in the community. The center also sends newsletters to survivors, caregivers, local clinics, and local clinicians to inform them of news, new events, and new projects. With this effort, the registry has been growing with > 10 new enrollees per month. In addition, trial information is available via internet (e.g., ClinicalTrials.gov, South Carolina Research Studies Directory).

### Assignment of interventions: allocation

#### Sequence generation {16a}

A computer-generated random allocation sequence will be used. Block randomization will be used to ensure balance (half in the experimental, half in the control). Block sizes will be randomly chosen between 8 and 38.

#### Concealment mechanism {16b}

At the beginning of each intervention session, the custom-developed software program is used to determine the sensory threshold. The program requires a participant number. Upon completion of the sensory threshold determination, the program will access the computer-generated random allocation sequence, find the assignment information for the participant number, and apply that group assignment information to the device’s vibration output. Therefore, the research personnel will not be involved in assigning groups and thus be blinded to the group assignment. Participants will be blinded, because TheraBracelet stimulation is subthreshold and both groups will not feel any vibration.

#### Implementation {16c}

The allocation sequence will be generated by the computer. The approved study staff will enroll participants. The custom-developed program will assign participants to the group according to the computer-generated allocation sequence.

### Assignment of interventions: blinding

#### Who will be blinded {17a}

Participants, care providers, intervention providers, outcome assessors, and data analysts except for the primary biostatistician will be blinded to the group assignment.

#### Procedure for unblinding if needed {17b}

A permission for unblinding will be deliberated and reviewed by the Data and Safety Monitoring Board (DSMB) if unanticipated intervention-related serious adverse events warrant investigation using the group assignment information.

### Data collection and management

#### Plans for assessment and collection of outcomes {18a}

The outcome assessment timeline is provided in Fig. 2. All clinical hand function tests (WMFT, BBT, and ARAT) will be administered by a blinded research therapist, videotaped, coded in names, and scored by raters who are blinded to the group assignment as well as timing of the videos (i.e., before or how many weeks after the intervention). Raters will be trained until excellent intra/interrater reliability is met with correlation greater than 0.9.

WMFT time has been validated for test-retest and interrater reliability in chronic stroke survivors [124, 125], and its minimal detectable change has been established [119]. WMFT time showed responsiveness to TheraBracelet treatment in our pilot study [54]. BBT and ARAT have also been validated for test-retest and interrater reliability [122, 126–128] and shown to be responsive to changes [112, 129] in stroke survivors. Minimal detectable change for BBT [122] and minimal clinically important difference for ARAT have been reported [115].

To gauge what participants perceive they can do functionally that they could not do before, we will obtain the Stroke Impact Scale hand and activities of daily living subscales [121, 130]. MAL [111] is used to assess the perceived quantity and quality of the paretic hand use in activities of daily living as part of the transfer package. The perceived meaningfulness of the intervention will be obtained on a 7-point Likert scale [115] (1 = much better, 2 = a little better, meaningful, 3 = a little better, not meaningful, 4 = about the same, 5 = a little worse, not meaningful, 6 = a little worse, meaningful, 7 = much worse) after completion of the intervention. Usability feedback [131] will also be obtained for TheraBracelet.

Accelerometers have been used to objectively capture changes in stroke survivors’ upper limb usage in daily living before and after therapy [132]. Although accelerometer data can be influenced by the whole body motion (e.g., walking) or non-purposeful upper limb movement, accelerometer data have been shown to significantly correlate with the number of observed purposeful repetitions [133–135] and adequately represent changes in the amount of functional upper limb use [114]. Tri-axial accelerometers (GT9X Link, ActiGraph, Pensacola, FL, USA) will be worn on each wrist for 3 days to capture upper limb activity of an average day [114]. Accelerometers will not interfere with TheraBracelet, as accelerometers are worn outside the laboratory and TheraBracelet is worn in the laboratory during therapy only. From 3-dimensional limb acceleration data recorded in 1 s bins over days [132], we will compute the duration and intensity of the paretic upper limb movement relative to the nonparetic limb (“use ratio” and “magnitude ratio,” respectively) [132, 135] as objective outcome measures.

Sensorimotor grip control will be quantified using the well-established biomechanical sensorimotor integration measures of digit force directional control [116, 117, 136–138] and efficient scaling of grip force [139–144]. Neural communication for hand grip will be quantified as EEG connectivity [118, 145] in the cortical sensorimotor network during the paretic hand grip. Accelerometer and sensorimotor grip control will be analyzed using a custom-scripted code in MATLAB (The MathWorks, Natick, MA, USA), and neural communication will be analyzed using Brainstorm [146] to obtain the final metrics by the blinded researcher post data collection.

#### Plans to promote participant retention and complete follow-up {18b}

To promote participant retention and complete follow-up, effective communication will be maintained between study staff and participants. Schedules, changes to schedules, and expectation for each visit will be clearly communicated.

#### Data management {19}

All electronic data will be stored in a password-protected secure research server. Visit records in paper will be scanned and stored in the password-protected secure research server. Data will be entered into a computer-based database. Quarterly data quality assessments will be performed by examining the outcomes databases for missing data, unexpected distributions or responses, irregularities, and outliers. Accuracy and completeness of the data collected will also be ensured.

#### Confidentiality {27}

The consent and HIPAA forms where personally identifiable information is recorded will be stored in a locked cabinet in a locked office. Only study personnel will have access to this personally identifiable information. For the video recording of the upper limb function tests, we will set the camera angle such that the video recording does not capture the participant’s face, while capturing the hand and arm movements and the interaction between the hand and objects in hand manipulation. All data will be coded with a participant code, and no personally identifiable information will be used to label the data. This means individual results would not be able to be linked to the participant by others who review the results of this research. De-identified paper data including testing sheets documenting testing sequences and notes will be stored in a cabinet in a key-locked room that is accessible to study personnel only. The linkage between the participant identities and participant codes will be stored in a locked cabinet in a locked room and will be accessible to study personnel only.

#### Plans for collection, laboratory evaluation, and storage of biological specimens for genetic or molecular analysis in this trial/future use {33}

This trial does not involve collecting biological specimens for storage.

### Statistical methods

#### Statistical methods for primary and secondary outcomes {20a}

The primary analysis will be a repeated measures general linear model with compound symmetry covariance structure (although other structures will be compared) for each outcome. The dependent variables will be the change from baseline at the evaluation times. The primary independent variables are group (treatment vs. control), evaluation time, and their interaction. We will also include sex as a biological independent variable along with its interactions to study sex difference. If the group×time interaction is significant, the main hypothesis that there is a group difference at follow-up will be tested using post hoc tests. Diagnostics will be performed on the residuals and appropriate actions will be taken if assumptions are not met. Summary statistics and graphs with a scatter plot matrix of the outcomes across time will be reviewed. Analyses will be performed using SAS© v.9.4. Greater increase in hand function for the treatment than control (with a significant group×time interaction) will support the hypothesis.

In secondary analysis, we will examine the time course of effects by analyzing the group differences in outcomes longitudinally (baseline at week 0, intervention week 1–6, post, and follow-up at 10 weeks) and finding the best fit regression model. This analysis will also inform the minimum duration to observe a significant effect. We will also test if the treatment group perceives the intervention more meaningful than the control group. An ordinal logistic regression assuming proportional odds will be considered.

#### Interim analyses {21b}

No interim analysis is planned. The DSMB may recommend stopping the study if the study has unanticipated safety concerns that warrant stopping.

#### Methods for additional analyses (e.g., subgroup analyses) {20b}

There will be no other additional analysis other than the analyses mentioned above.

#### Methods in analysis to handle protocol non-adherence and any statistical methods to handle missing data {20c}

We will use intent-to-treat analysis. If missing data arise, multiple imputation methods will be applied under the assumption of missing at random.

#### Plans to give access to the full protocol, participant level-data and statistical code {31c}

The protocol will be shared in ClinicalTrials.gov. De-identified participant-level dataset and/or statistical code will be shared upon reasonable request in writing.

### Oversight and monitoring

#### Composition of the coordinating center and trial steering committee {5d}

Study oversight will be provided by the DSMB. The DSMB will be composed of a board-certified stroke neurologist, a registered and licensed occupational therapist, and a biostatistician with expertise in design and analysis of clinical trials. The DSMB members will be experienced in care of stroke survivors and/or stroke recovery research. The DSMB will convene semiannually to review enrollment and study progression.

The trial management will be performed by the principal investigator, co-investigators, and the IRB-approved study personnel. The trial team will meet weekly or as necessary to discuss the trial setup, operation, progression, data analysis, interpretation, and dissemination. The principal investigator and study personnel are responsible for day-to-day operation and organization of the trial including identifying potential recruits and taking consent.

#### Composition of the data monitoring committee, its role and reporting structure {21a}

The DSMB will also ensure the safety of participants and the validity and integrity of data collected during the study. The DSMB will review adverse event data and provide a report to the IRB. The DSMB will be independent from the sponsor and competing interests.

#### Adverse event reporting and harms {22}

Adverse events will be solicited at each visit, recorded, and coded in terms of frequency, severity, relatedness to the intervention, and unanticipated nature using established guidelines [147–149]. All serious adverse events will be investigated by an independent medical monitor to determine relatedness to the intervention. The report by the independent medical monitor will be reviewed by the DSMB. All related serious adverse events will be reported to the IRB as they occur. All adverse event data will be tabulated and reported to the DSMB and ClinicalTrials.gov.

#### Frequency and plans for auditing trial conduct {23}

The sponsor will audit the study at random. The sponsor will review the study progression, regulatory compliance, and training compliance of all study personnel.

#### Plans for communicating important protocol amendments to relevant parties (e.g., trial participants, ethical committees) {25}

Any changes will be approved by the IRB prior to being in effect. Changes will be updated in ClinicalTrials.gov.

#### Dissemination plans {31a}

Trial results will be disseminated in ClinicalTrials.gov, in publications, and in conferences, and in-service/community presentations.

## Discussion

In case the sensory threshold changes over time, we will determine the participant’s sensory threshold at each intervention session for both groups. Participants might request wearing the device more than during therapy (e.g., at home) but will not be allowed based on the study need to control wear time. This research will determine clinical utility of TheraBracelet. If TheraBracelet is found efficacious, it is expected that TheraBracelet can be easily integrated in clinical practice. Enhancing hand function should increase stroke survivors’ independence and quality of life.

## Trial status

Protocol #6. July 28, 2021. Recruitment began November 2, 2020, and is expected to conclude in November 2025.

## Abbreviations

ARAT: Action Research Arm Test
AR(1): Autoregressive
BBT: Box and Block Test
DSMB: Data and Safety Monitoring Board
EEG: Electroencephalogram
GABA: Gamma aminobytyric acid
IRB: Institutional Review Board
MAL: Motor Activity Log
MCID: Minimal clinically important difference
MUSC: Medical University of South Carolina
NIH: National Institutes of Health
RESTORE: MUSC Registry for Stroke Recovery
SC: South Carolina
SD: Standard deviation
TENS: Transcutaneous electrical nerve stimulation
TMS: Transcranial magnetic stimulation
USA: United States of America
WMFT: Wolf Motor Function Test
